# Shu-Di-Huang and Gan-Cao Herb Pair Restored the Differentiation Potentials of Mesenchymal Stem Progenitors in Treating Osteoporosis via Downregulation of NF-*κ*B Signaling Pathway

**DOI:** 10.1155/2021/7795527

**Published:** 2021-12-14

**Authors:** Xiaopeng Ling, Xiaojian Wang, Jinghu Li, Taiwei Zhang, Xing Zhou, Yiyuan Zhou, Jian Kang

**Affiliations:** ^1^Department of Orthopaedics, The Fourth Affiliated Hospital of Nanchang University, Nanchang, Jiangxi, China; ^2^The First Clinical College, Zhejiang Chinese Medical University, Hangzhou, Zhejiang, China; ^3^Department of Tuina, The Third Affiliated Hospital of Zhejiang Chinese Medical University, Hangzhou, Zhejiang, China; ^4^Obstetrics Department, The First Affiliated Hospital of Guizhou University of Chinese Medicine, Guiyang, Guizhou, China; ^5^Department of Orthopaedics, Shanghi University of Traditional Chinese Medicine Taizhou Hospital, Taizhou, Zhejiang, China; ^6^Department of Orthopaedics, The Affiliated Hospital of Jiangxi University of Chinese Medicine, Nanchang, Jiangxi, China; ^7^Jiangxi University of Chinese Medicine, Nanchang, Jiangxi, China

## Abstract

**Background:**

Shu-Di-Huang (Radix Rehmanniae Praeparata, RR) and Gan-Cao (liquorice, L) are frequently used traditional Chinese herb pair in treating osteoporosis (OP). However, the exact mechanism of the RR and L herb pair (RR-L) remains unclear. To explore the efficacy and possible mechanisms of RR-L in treating OP, in silico, in vitro, and in vivo experiments were conducted in the current study.

**Methods:**

In silico, potential therapeutic target genes and active chemical compounds of RR-L herb pair were predicted and constructed into a network. In vivo, 30 Sprague Dawley rats were divided into 3 groups, including the sham group, the OP model group, and the RR-L-treated OP group. Micro-CT and pathological sections were conducted to validate the therapeutic effects of RR-L in treating OP. MSCs of rats were isolated and cultured in vitro to validate the mesenchymal stem cells (MSCs) related phenotype changes, including Alizarin red staining, Oil red staining, and immunofluorescence. In vitro, cell proliferation analysis, Alizarin red staining, Oil red staining, immunofluorescence of NF-*κ*B, and protein expression of PPAR*γ*, RUNX2, OCN, and p65 were conducted on MSCs to explore the RR-L containing serum in vitro. Also, activator and inhibitor of NF-*κ*B signaling pathway were introduced to determine the possible mechanism of RR-L in the treatment of OP via enhancing MSCs proliferation and differentiation.

**Results:**

In silico, 168 chemical compounds with a property of oral bioavailability ≥30% and drug-likeness ≥0.18 were recognized as potentially active compounds in RR-L and 249 genes were found to be the targets of which. Among them, 120 genes were found to be therapeutic genes of RR-L in treating OP and KEGG and GO analysis of which demonstrated that RR-L involves in lipid metabolism and multiple inflammation-related signaling pathways. In vivo, ovariectomy- (OVX-) induced OP phenotypes in Sprague Dawley rats include bone mineral density and microarchitecture damaging, abnormal bone metabolism, upregulation of inflammation markers, and damaged differentiation potential of MSCs. Treatment of RR-L reversed the trend and restored the differentiation potential of MSCs. In vitro, RR-L containing serum promoted the osteogenic differentiation and suppressed adipogenic differentiation of MSCs via downregulation of the NF-*κ*B signaling pathway. Also, RR-L containing serum inhibited the tumor necrosis factor-*α* (TNF-*α*) induced activation of the NF-*κ*B signaling pathway. On the opposite, the addition of the NF-*κ*B specific inhibitor significantly reduced the effect of RR-L on MSCs.

**Conclusions:**

In the current study, network pharmacology prediction and experimental validation elucidated that the RR-L herb pair restored damaged MSC differentiation potential via the NF-*κ*B signaling pathway; this could be the possible mechanism of RR-L in treating OP. This finding provides an alternative option in OP therapy.

## 1. Background

Osteoporosis (OP) is the most common bone metabolic disease, causing a rapidly increasing burden of public health in middle and aging populations worldwide [1]. It is reported that the prevalence of OP was 6.46% and 29.13% for elderly men and women, respectively [2]. OP is characterized by bone mineral density (BMD) loss, bone microarchitecture degenerated, and risk of fracture upregulated [3]. It is well accepted that OP is led by an imbalance between bone formation and bone resorption [4, 5]. First-line clinical therapies mainly focused on inhibition of excessive bone resorption activity and few drugs focusing on enhancing attenuated bone formation were developed. Therefore, it is necessary to explore more alternative options in OP therapy.

Mesenchymal stem cells (MSCs) are a subgroup of bone marrow stromal cells with the typical ability of self-renewal and multiple lineage differentiation potentials which contribute to osteoblasts, adipocytes, chondrocytes, and stromal cells. In most strains or animal models, the differentiation commitment toward different lineages holds rigorously inversely related, as the osteogenic differentiation of MSCs requires a regulated inhibition of adipogenesis differentiation [6]. Consistent with this inverse relation of lineages, previous studies involved in clinical human studies reported the increasing risk of fracture correlated with decreasing bone mineral density and high bone marrow fat content, especially in OP patients [7]. Many efforts explored the origin of the imbalance of osteoblastic and osteoclastic activation and developed valid strategies of inhibiting the abnormal formation and function of osteoclasts, including bisphosphate and anti-RANKL antibody [8]. Recently, emerging attention has been attracted to other aspects of bone coupling, involving aberrant lineage differentiation of MSCs. Increased bone marrow adipose tissue accumulation caused impaired bone formation and regeneration. The differentiation fate decisions of MSCs were rigorously regulated by molecular signals and cues within the cellular microenvironment, and several transcriptional cellular cytokines have been identified [9]. Runx2, OCN, and Osterix were identified to be the main regulators of osteogenic differentiation [10]. PPAR*γ* was viewed as the essential role of adipogenesis progression [10]. However, the upstream mechanism regulating the MSC differentiation behaviors is not fully elucidated. It is particularly essential to explore the molecular mechanism when emerging strategies focus on rectifying the aberrant differentiation and allocations of MSCs related to OP pathology. Emerging interests were attracted to natural products for their therapeutical potential in treating OP, especially in alleviating damaged osteogenic differentiation [11].

Traditional Chinese medicine (TCM) had attracted much attention for its efficacy and safety in OP treatment [12–14]. Previous studies demonstrated multiple TCM formulas or decoctions to be effective in OP treatment [13, 15]. However, the complexity of the TCM formula impeded the full understanding of its mechanism in OP therapy. Also, the TCM formula often consisted of multiple herbs, which made it extremely difficult to optimize the compose of the formula. According to our knowledge and previous studies, Shu-Di-Huang and Gan-Cao herb pair (RR-L herb -pair) seems to be an effective and frequently used herb pair of TCM formula in OP treatment. To explore the effect and mechanism of RR-L herb pair in treating OP, a combined strategy of network pharmacology and experimental validation was conducted.

In the current study, “herb-compounds-targets” and enrichment analysis of RR-L herb pair in treatment of OP were conducted in silico, and results demonstrated that RR-L involves in multiple inflammation-related signaling pathways and could possibly regulate lipid metabolism. In experimental validation, RR-L herb pair was given to OVX-induced OP rats to evaluate its efficacy and influence on OP treatment. In vivo studies demonstrated RR-L successfully reversed OVX-induced alterations of MSCs, BMD loss, and microarchitecture damage. In vitro, RR-L enhanced the MSC osteogenesis differentiation potential in a dose-dependent manner. Also, with inhibitor and activator of NF-*κ*B signaling pathway, it has been found that RR-L regulates the MSCs differentiation behavior via NF-*κ*B signaling pathway.

## 2. Methods and Materials

### 2.1. Network Pharmacology Prediction

In the current study, the network pharmacology analysis method refers to the previous study. We followed the methods of [5]. The chemical components of TCM herbs of Radix Rehmanniae Preparata and liquorice were searched on TCMSP and SYMMAP databases [16, 17]. The search term was set to be “Shu-Di-Huang” and “Gan-Cao.” The search was done on March 19^th^, 2020. The criteria of active chemical compounds were set to be oral bioavailability (OB) ≥30% and drug-likeness ≥0.18. After screening, the potential target genes of these chemical compounds were collected from the databases. OP-related disease genes were collected from GeneCards and OMIM databases [18, 19]. The search term was set to be “osteoporosis.” The search was done on March 19th, 2020. The resulting OP-related disease genes were collected and combined, followed by removing the duplications. The intersection of RR-L target genes and OP-related disease genes was identified by Venn analysis by R software and packages. Visualized network construction of “chemical compounds-target gene” was conducted by Cytoscape software. Hub gene analysis was conducted with the Cytoscape plug-in CytoHubba and ranked by the degree value of the gene network. Protein-protein interaction (PPI) network was done on the STRING database [20]. Gene ontology (GO) analysis and KEGG enrichment analysis were conducted on the DAVID database through R software and packages [21].

### 2.2. RR-L Herb Pair and RR-L Containing Serum Preparation

RR-L, composed of Shu-Di-Huang 15 g and Gan-Cao 3 g, was soaked in four times volume of water and boiled for 30 min, filtrated twice. We collected the filtrated herb liquor and concentrated and dried it to RR-L extraction. Approximately 3 g dry RR-L extractions were collected per 15 g RR-L crude herbs. Extractions were diluted and stored at 4°C. For in vitro experiments, RR-L extractions were given to 10-week-old female Sprawl–Dawley rats for 7 days (with a dosage of 0.25 g/kg); after treatment, the rats were sacrificed and the blood sample was collected through the abdominal aortic method. After centrifuge and sterilization, the RR-L containing blood serum was collected and stored at −20°C for further studies.

### 2.3. LC-MS Analysis of RR-L Herb Pair

200 mg RR-L extraction was added with 1 ml methanol, whirled for 10 min, centrifuged at 4°C for 10 min, filtered with a 0.22 *μ*m filter membrane, and subjected to Ultimate 3000 RS system (Thermo Fisher Scientific, MA, USA) equipped with a Thermo Hypersil GOLD column (*φ* 2.1 × 100 mm, 1.9 *μ*m). The MS spectra were acquired by a Q Executive high-resolution mass spectrometer (Thermo Fisher Scientific, MA, USA). The mobile phases were (A) 0.1% formic acid in water (B) and 0.1% formic acid in acetonitrile, and the gradient elution program was as follows (time/B%): 0–1 min, 2%; 1–5 min, 2%–20%; 5–10 min, 20–50%; 10–15 min, 50–80%; 15–20 min, 80–95%; 20–25 min, 95%; 26–30 min, 2%. The chromatographic analysis was performed at 35°C with a flow rate of 0.3 mL/min and an injection volume of 15 *μ*L. The mass spectrometer parameters were as follows: spray voltage was set at 3.8 kV at positive mode. The capillary temperature was set at 300°C. Argon was used as the collision gas. Nitrogen was used as sheath gas and aux gas. Aux gas heater temperature was set to be 350°C. The chromatogram was analyzed by CD 2.1 software.

### 2.4. Cell Isolation and Culture

MSCs were obtained from the bone marrow cavity of femoral bone and tibia bone of rats housed in the Laboratory Animal Science and Technology Centre of Jiangxi University of TCM (Nanchang City, Jiangxi Province, China). Femoral bone and tibia bone were collected and washed with DMEM through injectors. The resulting cells were cultured on the dishes, and the suspension cells were removed through medium change after 90 min. Then, the medium was changed every 24 h, digested with 0.25% trypsin (Solarbio, Beijing, China), and passaged to new dishes, and the suspension cells were removed after passage. The resulting MSCs were cultured in 25 cm^2^ culture flasks with DMEM (Solarbio, Beijing, China) containing 10% FBS (Gibco, NY, United States) and antibiotics (100 U/ml penicillin, 0.1 mg/ml streptomycin) in 5% CO_2_ at 37°C conditions.

mscMSCs were seeded at 6-well plates at an initial density of 5 × 105 cells/well and then serum starved for 12 h. MSCs cells were treated with a series of RR-L containing serum concentrations with or without TNF-*β* for further studies in osteogenesis medium or adipogenesis medium. Alternatively, MSC cells were incubated with 15% RR-L containing serum with or without BAY117082 for further analysis in osteogenesis medium or adipogenesis medium.

### 2.5. MTT Assay

mscMSCs were cultured in 96-well plates at about 5 × 103 cells in each well and treated with various concentrations of RR-L containing serum (0%, 5%, 10%, 15%, and 20%) for various time periods (24 and 48 h), respectively. Six duplications were applied for each well. Six wells without RR-L containing serum and another six wells without cells were used as the control and blank, respectively. 5 mg/ml MTT was mixed with the culture medium at a concentration of 20 *μ*l for each well at 37°C for 4 h. The supernatant was removed, and then 150 *μ*l DMSO was added per well. Optical density was measured at 570 nm with a multimode reader (Spark 10 M, TECAN, Switzerland).

### 2.6. Alizarin Red Staining

mscMSCs were seeded at a 6-well plate. Cells were washed and followed by a culture medium (consisting of 10% FBS, 50 *μ*M ascorbic acid, 10 mm glycerine *β*-phosphate, 100 nm dexamethasone in *α*-MEM culture medium) for induction of osteogenic differentiation for 21 days. The medium was changed every 72 h. The cells were fixed in 10% methanol at room temperature after air-drying and left for 30 min at −20°C. Cells were stained with 1 ml/well 40 mM Alizarin red S (Solarbio, Beijing, China) at room temperature. After being washed, cells were observed using a Leica Confocal 222 microscope.

### 2.7. Oil Red Staining

mscMSCs were seeded at a 6-well plate. MSCs were washed and followed by adipogenesis inducing culture medium (consisting of 10% FBS, 200 *μ*M indomethacin, 0.5 mM IBMX, 10 *μ*g/ml insulin, 1 *μ*m dexamethasone in HG-MEM culture medium) for induction of osteogenic differentiation for 14 days. The medium was changed every 48 h and followed by 10 *μ*g/ml insulin medium for 24 h. The cells were fixed in 27.5% methanol at room temperature after air-drying and left for 30 min at −20°C. Cells were stained with 0.5% Oil Red staining (Solarbio, Beijing, China) at room temperature. After being washed with 70% ethanol, cells were observed by Leica Confocal 222 Microscope.

### 2.8. Immunofluorescence

mscMSCs were seeded at a 6-well plate, washed with PBS 3 times, fixed for 15 min followed by PBS wash, treated with 0.5% triton for 20 min at room temperature, blocked with goat serum at room temperature for 30 min followed by incubation with primary anti-p65 antibody overnight, added to fluorescent secondary antibodies, stained with DAPI for 5 min, sealed with an antifade solution, and observed by Leica Confocal 222 Microscope.

### 2.9. Western Blot

Protein samples were separated by sulfate-polyacrylamide gel electrophoresis and then blotted onto polyvinylidene fluoride membranes. The proteins on the blot were assessed using primary antibodies against RUNX2 (Abcam, ab194726), OCN (Thermo, MA1-20786), PPAR*γ* (Abcam, ab272718), p65 (Abcam, ab76956), and *β*-actin followed by HRP-conjugated goat anti-mouse antibody or anti-rabbit secondary antibody. Blots were visualized using the electro-chemiluminescence method. The density of protein bands was quantified with Image Lab software.

### 2.10. RT-PCR

Total cellular RNA was isolated using TRIzol reagent (Invitrogen, Carlsbad, USA) according to the manufacture's instruction. Complementary DNA (cDNA) was reverse-transcribed with a PrimeScript-RT reagent kit (TaKaRa Biotechnology Co, Ltd., Japan). The mRNA levels of RUNX2, OCN/BGLAP, PPAR*γ*, p65/RELA, and 18s RNA were detected by quantitative real-time PCR with SYBR Premix Ex Taq kit (TaKaRa Biotechnology Co, Ltd., Japan). Primer sequences used are shown in Supplementary Table 3 and the specificity of sequences was verified using the BLAST algorithm of the National Centre for Biotechnology Information. Data were normalized to 18 s rRNA and analyzed by the 2^(−ΔΔCT)^ method.

### 2.11. Animal Study

30 female Sprague Dawley rats (200 ± 20 g) were purchased from the Laboratory Animal Science and Technology Centre of Jiangxi University of Traditional Chinese Medicine (Nanchang City, Jiangxi Province, China). All rats were free to access diet and water. All animal care and protocols were approved by the Committee of Management and Use of Laboratory Animals of Jiangxi University of Traditional Chinese Medicine (Nanchang City, Jiangxi Province, China). All animal experiments complied with the Guide for the National Institutes of Health guide for the care and use of laboratory animals.

Briefly, all rats except the sham group were anesthetized by the intraperitoneal injection of pentobarbital, and skin was prepared. Rats were fixed in the supine position for surgery. After general anesthesia, the operation area was disinfected, and the towels were spread. A longitudinal incision was made, and the skin and subcutaneous tissue were incised. The abdomen was opened, and the uterus was located and pulled out of the abdominal cavity. The ovary was cut off. The same procedure was performed on the opposite side to excise both ovaries. As for the sham group, the ovaries of rats were reserved, and only similar weight fat to the ovary was excised. All rats were randomly divided into 3 groups equally: (1) sham-operated, (2) bilaterally ovariectomies (OVX), and (3) OVX rats treated with RR-L. Blood samples were collected for enzyme-linked immunosorbent assay (ELISA). All rats were sacrificed after 12 weeks of treatment. Femur tissue was collected for further analyses.

### 2.12. Micro-CT Scanning and 3-Dimension Remodeling

Micro-CT scan (Skyscan 1176, Bruker micro-CT N.V, Kontich, Belgium) and 3-dimension remodeling were applied for radiographic observation. The right distal femur was harvested and cut to fit the appropriate size for micro-CT scan. Bone volume/Trabecular Volume (BV/TV), Trabecular separation (Tb. Sp), number of trabecular (Tb. N), and bone mineral density (BMD) were calculated by analysis software version 1.1.1.

### 2.13. ELISA Analysis

We followed the methods of [22]. Briefly, in vivo, samples of rat plasma were collected. The supernatant liquid was collected after centrifugation. These samples were used for ELISA assay for P1NP, ALP, IL-1*β,* and TNF-*α*, according to the manufacturer's instructions. Rat plasma was added to the 96-well plate and followed by incubation at 37°C. Washed with distilled water, an enzyme reagent was added followed by a chromogenic agent. Optical density was measured at 450 nm with a multimode reader (Spark 10M, TECAN, Switzerland).

### 2.14. Statistical Strategy

Data were presented as mean ± standard deviation and analyzed using SPSS 19.00 software. One-way analysis of variance (ANOVA) was introduced. *P* value <0.05 was considered statistically significant, and the exact *P* value was shown in respective figures.

## 3. Results

### 3.1. Inflammation, Bone Formation, and Lipid Regulation Related Genes and Pathways Were Found to Be the Targets of RR-L Herb Pair in Treating OP

**Figure 1:**
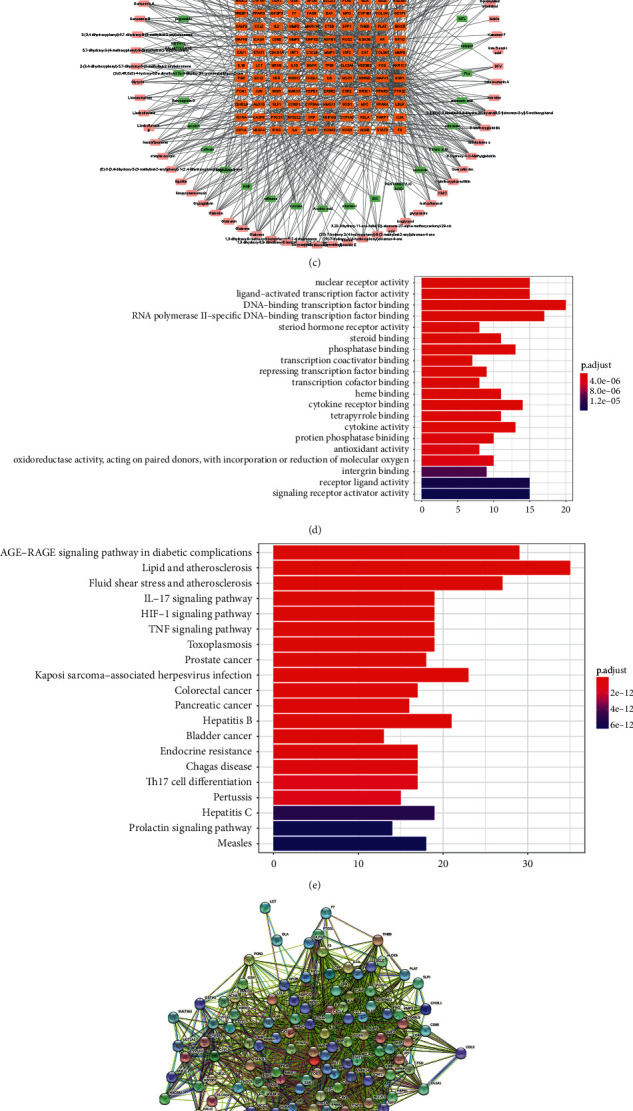
Network pharmacology analysis of RR-L in treating osteoporosis indicated RR-L involves multiple pathways including inflammation and lipid metabolism. (a) Herb-compounds-targets network construction. (b) Venn analysis revealed 120 shared genes between RR-L and OP. (c) Herb-compounds-OP targets network construction. (d) GO enrichment analysis of 120 shared target genes. (e) KEGG pathway enrichment analysis of 120 shared target genes. (f-g) PPI network and hub gene analysis of 120 shared target genes.

168 different chemical compounds were viewed as active compounds of RR-L herb pair according to the criteria of DL ≥ 0.18 and OB ≥ 30%, and 249 genes were found to be the targets of these 168 RR-L herb pair containing chemical compounds, as shown in Figure 1(a) (detailed data was shown in Supplementary Table 1). 120 genes were found to be the intersection of RR-L herb pair target genes and OP-related disease genes through Venn analysis, which demonstrated that these 120 genes might be the targets of RR-L herb pair in treating OP, as shown in Figure 1(b) (detailed data was shown in Supplementary Table 2). The compounds-targets network was constructed in Figure 1(c) followed by GO analysis and KEGG analysis. Results demonstrated RR-L herb pair is mostly involved in lipid metabolism-related signaling pathways and multiple inflammation-related signaling pathways including the IL-17 signaling pathway, HIF-1 signaling pathway, and TNF signaling pathway, as shown in Figures 1(d)–1(e). Also, the PPI network of the 120 intersection genes was conducted and shown in Figure 1(f). As shown in Figure 1(g), the hub gene analysis of the PPI network revealed that inflammation-related genes including JUN, MAPKs, HSPB1, and HIF1A were found to be the hub genes (detailed data was shown in Supplementary Table 2). Also, bone formation marker RUNX2 was also found to be the hub gene of the PPI network. In conclusion, regulation of inflammation, bone formation, and lipid metabolism might be the mechanism of RR-L herb pair in treating OP.

### 3.2. RR-L Ameliorated Bone Mineral Density Loss and Microarchitecture Damage in OVX-Induced OP Rats via Downregulation of Inflammation Markers In Vivo

**Figure 2:**
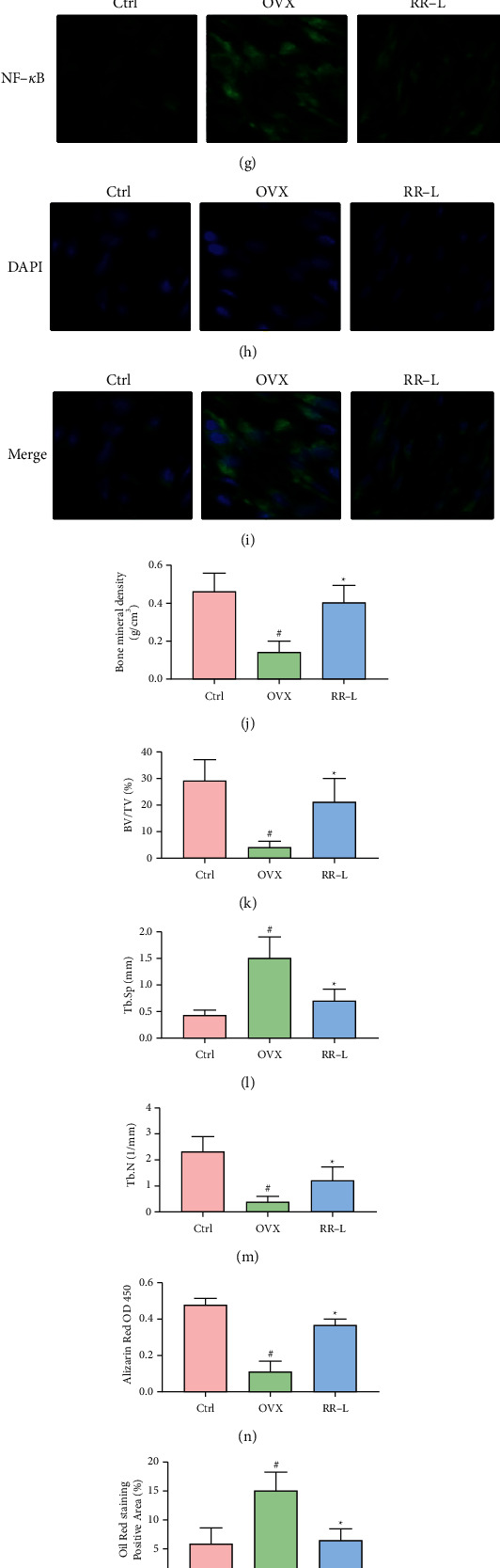
RR-L treatment reversed OVX-induced OP phenotypes in vivo. (a-b) Micro-CT and 3D remodeling of the femoral bone. (c-d) Pathological section staining including HE staining and toluidine blue staining. (e) Alizarin red staining of MSCs derived from OVX-induced OP rats. (f) Oil red staining of MSCs derived from OVX-induced OP rats. (g–i) Immunofluorescence analysis detects the activation of the NF-*κ*B signaling pathway in MSCs derived from OVX-induced rats. (j–m) Structural index including bone mineral density, BV/TV, Tb.Sp, and Tb.N obtained from (A-B). (n) Statistical result of (e) via Image J software. (o) Statistical results of (F) via Image J software. (p) Statistical results of (g–i) via Image J software. (q–t) ELISA analysis of serum P1NP, ALP, IL-1*β,* and TNF-*α*. ^#^*p* < 0.05 versus sham, ^*∗*^*p* < 0.05 versus control.

For further validation of the possible mechanism of RR-L in treating OP, OVX rats were induced as the OP animal model. Meanwhile, the RR-L solution used for in vivo tests was conducted with LC-MS analysis, and the results are shown in Supplementary Figure 1. As shown in Figures 2(a)-2(b), *μ*CT scanning and 3-dimension remodeling demonstrated that bone volume and bone microarchitecture were found to be significantly damaged in OVX-induced OP rats, compared to the sham group. According to the *μ*CT scanning results, the quantitative analysis of the bone volume and bone microarchitecture parameters including BMD, Tb. Sp, Tb. N, and BV/TV was conducted by CTAn software. As shown in Figures 2(j)–2(n), results demonstrated that OVX-induced OP rats possessed OP phenotypes including BMD loss and trabecular bone which is sparse, decreased, and thickened, compared to the sham group. Treatment of RR-L for 2 months significantly reversed the trend compared to the OVX group. In the RR-L group, the trabecular bone density increases and uniforms compared to the OVX group. Also, BMD was upregulated in the RR-L group compared to the OVX group.

As shown in Figures 2(c)–2(d), OVX-induced rats showed that trabecular bone is sparse and decreased combined with the bone marrow mesenchymal cell loss and vacuoles formation compared to the sham group. Compared to the OVX group, treatment of RR-L for 2 months restored the number and thickness of trabecula bone and also reduced the number and size of vacuoles in the bone marrow. HE staining and toluidine staining demonstrated that OVX impairs the resident cell group in the bone marrow and induced lipid vacuole formation and RR-L treatment reversed the trend. Also, as illustrated in Figures 2(q)–2(t), serum bone formation markers of ALP and P1NP were significantly downregulated, and serum inflammation markers of IL-1*β* and TNF-*α* were significantly upregulated in OVX-induced OP rats, compared to the sham group. RR-L treatment significantly improved the serum ALP and P1NP expression and decreased serum IL-1*β* and TNF-*α*, compared to the OVX group.

In conclusion, in vivo, RR-L reversed OVX-induced OP phenotypes including BMD loss, microarchitecture damage, bone marrow mesenchymal cell loss, lipid vacuole formation, decreased osteogenesis, and abnormal high level of inflammation, which illustrated that RR-L could possibly exert its anti-OP effects via suppression of inflammation and restored the bone marrow mesenchymal cells group.

### 3.3. RR-L Improved the Osteogenesis Differentiation and Decreased the Adipogenic Differentiation of MSCs Derived from OVX Rats Possibly via NF-*κ*B Signaling Pathway In Vitro

For validation of differentiation potential of MSCs, Alizarin red staining and Oil red staining were introduced to validate the osteogenesis and adipogenesis potential of MSCs, respectively. MSCs obtained and derived from OVX rats were subsequently cultured in vitro and conducted with Alizarin red staining and Oil red staining. As shown in Figures 2(e) and 2(n), MSCs derived from the OVX-induced OP rats' group significantly decrease the osteogenesis potential compared to the BMSCs derived from the sham group. MSCs derived from RR-L-treated OVX-induced rats showed enhanced osteogenesis potential compared to the OVX group. As shown in Figures 2(f) and 2(o), Oil red staining results illustrated that MSCs derived from OVX-induced rats possess a higher adipogenesis potential compared to the MSCs derived from the sham group, and MSCs derived from the RR-L group significantly inhibited the enhanced adipogenesis potential induced by OVX. Furthermore, to validate the potential interaction between inflammation and differentiation potential of MSCs, the nuclear translocation of p65, a downstream marker of inflammation-related signaling pathway NF-*κ*B, was detected by IF, as shown in Figures 2(g)–2(i). The statistical result, as shown in Figure 2(p), demonstrated that the NF-*κ*B signaling pathway was significantly activated in MSCs derived from OVX-induced OP rats compared to the sham group. NF-*κ*B signaling pathway was significantly downregulated in RR-L-treated OVX rats compared to the OVX group. In conclusion, RR-L treatment restored the impaired differentiation potential via downregulation of NF-*κ*B in MSCs induced by OVX.

### 3.4. RR-L Containing Serum Improved Differentiation Potential of MSCs via Inhibition of NF-*κ*B Signaling Pathway in a Dose-Dependent Manner In Vitro

**Figure 3:**
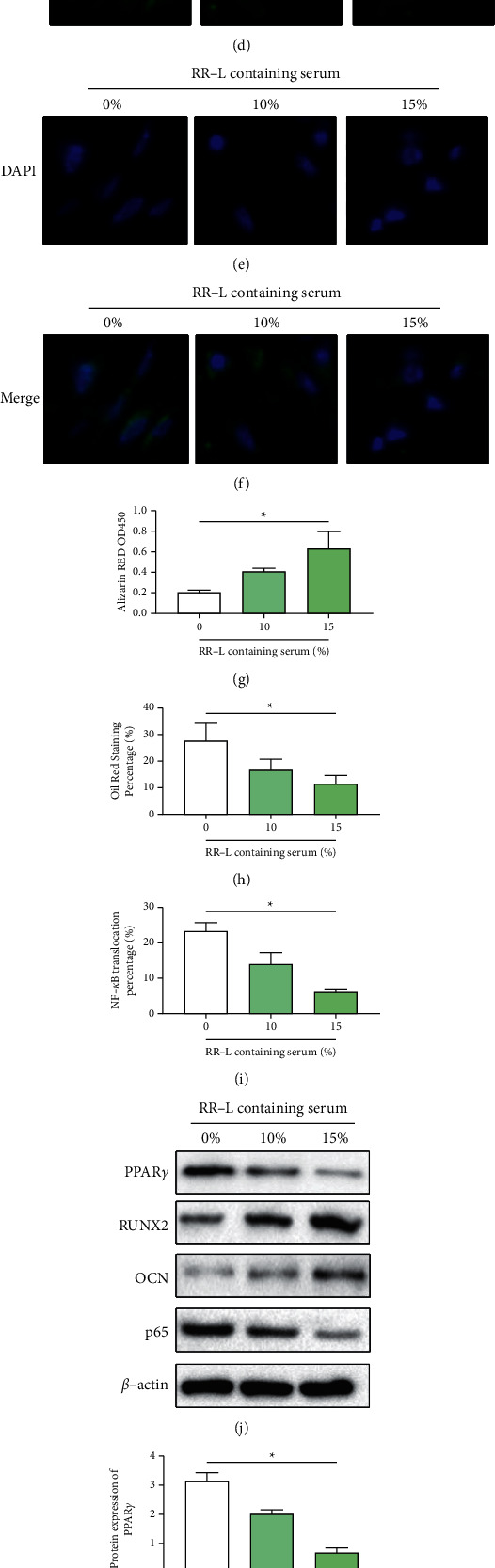
RR-L containing serum improved osteogenesis activity and suppressed adipogenesis activity in MSCs via inhibiting NF-*κ*B signaling pathway in a dose-dependent manner in vitro. (a) MTT assay revealed that 10%–15% RR-L containing serum promoted MSC proliferation in a dose-dependent manner. (b) Alizarin red staining revealed that RR-L containing serum improved osteogenesis activity in a dose-dependent manner. (c) RR-L containing serum suppressed adipogenesis activity in a dose-dependent manner. (d–f) RR-L containing serum inhibited NF-*κ*B signaling pathway in a dose-dependent manner. (g) Statistical result of (b). (h) Statistical result of (c). (i) Statistical result of (d–f). (j) Representative western blots image of PPAR*γ*, RUNX2, OCN, p65, and *β*-actin. (k–n) Statistical results of western blot via Image Lab software. ^*∗*^*p* < 0.05 versus control.

RR-L containing serum was prepared as illustrated in the Methods section. To determine the effects of RR-L containing serum on the proliferation of MSCs, an MTT assay was conducted. As shown in Figures 3(a)-3(b), cotreatment of RR-L containing serum improved the proliferation of MSCs in a dose-dependent manner from 10% to 15% for 24 h and 48 h. Mild inhibition of 20% RR-L containing serum on the proliferation of MSCs was observed. Meanwhile, no significant differences were found between 24 h and 48 h groups. Therefore, cotreatment of 10% and 15% RR-L containing serum was introduced in further study. As shown in Figures 3(b) and 3(g), Alizarin Red staining results demonstrated that 10% and 15% RR-L containing serum significantly increased the osteogenesis potential of MSCs. Consistent with the Alizarin red staining, protein expression of osteogenesis biomarkers of RUNX2 and OCN, as shown in Figures 3(j) and 3(l)-3(m), illustrated that 10% and 15% RR-L containing serum significantly increased the osteogenesis differentiation level in a dose-dependent manner. Oil red staining results, as shown in Figures 3(c) and 3(h), illustrated that 10% and 15% RR-L containing serum significantly decreased adipogenesis potential of MSCs. Also, the protein expression of adipogenesis biomarker PPAR*γ* was detected. As shown in Figures 3(j) and 3(k), consistent with the Oil red staining result, 10% and 15% RR-L containing serum decreased the adipogenesis biomarker in a dose-dependent manner. To determine whether NF-*κ*B is involved in the regulation of RR-L containing serum in treating MSCs, the nucleus protein expression and nucleus translocation of p65 were conducted by western blot and immunofluorescence, respectively. As shown in Figures 3(d)–3(f), the nucleus translocation of p65 of MSCs was significantly decreased with the cotreatment of 10% and 15% group in a dose-dependent manner. Meanwhile, as shown in Figures 3(j) and 3(n), the nucleus protein expression of p65 was significantly decreased by 10%–15% RR-L containing serum in a dose-dependent manner. In conclusion, the RR-L herb pair significantly promoted MSCs proliferation, increased the osteogenesis potential, and decreased the adipogenesis potential via inhibiting activation of the NF-*κ*B signaling pathway in a dose-dependent manner. Also, 15% RR-L containing serum has the best effect on MSCs in the current study in vitro.

### 3.5. TNF-*α* Induced Activation of NF-*κ*B Mediated Differentiation Potential Damage of MSCs Was Reversed by Cotreatment of RR-L Containing Serum In Vitro

**Figure 4:**
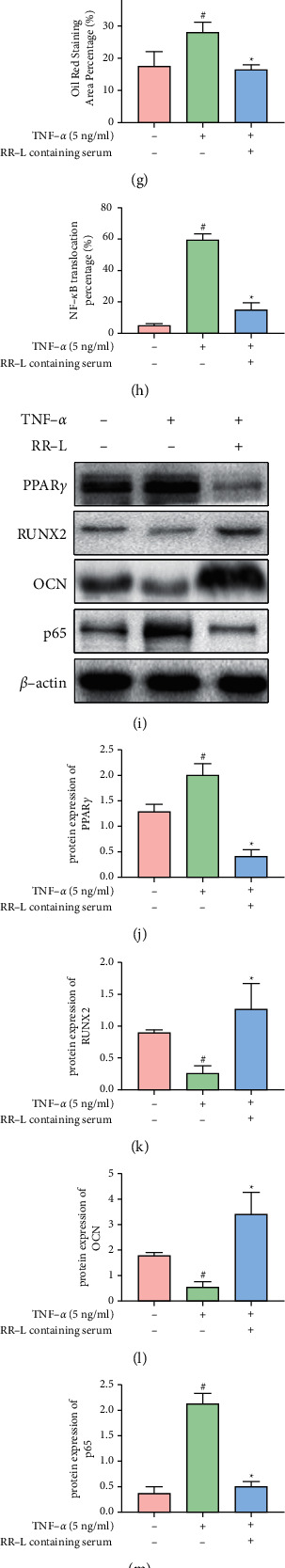
RR-L reversed TNF-*α*-induced NF-*κ*B signaling pathway activation related to MSC differentiation alterations. (a) RR-L containing serum inhibited TNF-*α*-induced osteogenesis inhibition of MSCs. (b) RR-L containing serum inhibited TNF-*α*-induced adipogenesis activation of MSCs. (c–e) RR-L containing serum inhibited TNF-*α*-induced NF-*κ*B signaling pathway activation of MSCs. (f–h) Statistical results of Alizarin red, Oil red staining, and IF analysis. (i) Representative western blots image of PPAR*γ*, RUNX2, OCN, p65, and *β*-actin. (j–m) Statistical results of western blot via Image Lab software. (n–q) mRNA expressions of PPAR*γ*, RUNX2, OCN, and p65. ^#^*p* < 0.05 versus ctrl, ^*∗*^*p* < 0.05 versus TNF-*α*.

For further understanding of the role of NF-*κ*B in the differentiation of MSCs, TNF-*α* was introduced as an agonist of NF-*κ*B on MSCs. As shown in Figures 4(c)–4(e), TNF-*α* significantly improved the nucleus translocation of p65 on MSCs, which means successfully activated NF-*κ*B signaling pathway. Along with the activation of the NF-*κ*B signaling pathway, a significant decrease of osteogenesis potential was found in Alizarin red staining and protein expression of osteogenesis biomarkers of RUNX2 and OCN. Adipogenesis level was found to be significantly increased via Oil red staining detection and adipogenesis biomarker of PPAR*γ*, which means that TNF-*α* induced activation of NF-*κ*B signaling pathway in MSCs presented similar cellular phenotype alterations in MSCs derived from OVX-induced rats. Cotreatment with RR-L containing serum significantly reversed the alterations induced by TNF-*α*. RR-L containing serum upregulated the osteogenesis level compared to the TNF-*α* group, as shown in Figures 4(a) and 4(f). Oil red staining results demonstrated that RR-L containing serum significantly inhibited TNF-*α* induced adipogenesis differentiation of MSCs, as shown in Figures 4(b) and 4(g). Also, gene and protein expressions of osteogenesis biomarker of RUNX2 and OCN were detected, respectively, as shown in Figures 4(i), 4(k)-4(l), and 4(o)–4(p), demonstrating that RUNX2 and OCN were upregulated in the cotreatment of RR-L containing serum compared to TNF-*α* induced MSCs. The gene and protein expressions of adipogenesis biomarkers of PPAR*γ* were also conducted, and results demonstrated that RR-L containing serum inhibited TNF-*α* induced upregulation of PPAR*γ*, as shown in Figures 4(i)-4(j) and 4(n). The nucleus translocation of p65, as shown in Figures 4(c)–4(e) and 4(h), demonstrated that RR-L containing serum significantly inhibited TNF-*α* induced accumulation and nucleus translocation of p65. The nucleus protein and gene expression of p65 also demonstrated that RR-L containing serum inhibited the activation of the NF-*κ*B signaling pathway, as shown in Figures 4(i), 4(m), and 4(q). In conclusion, TNF-*α* induced activation of NF-*κ*B signaling pathway manifested damaged differentiation potential similar to the alterations of MSCs in OVX-induced OP rats and RR-L containing serum significantly reversed TNF-*α* induced alterations via NF-*κ*B signaling pathway.

### 3.6. Cotreatment of NF-*κ*B Signaling Pathway Inhibitor BAY117082 and RR-L Containing Serum Showed No Better Effect than BAY117082 Along on MSCs In Vitro

**Figure 5:**
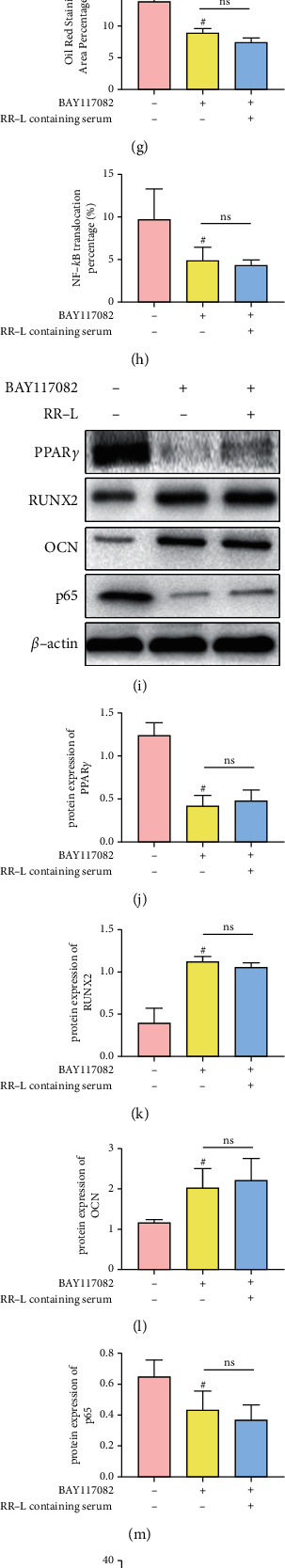
Cotreatment of NF-*κ*B signaling pathway inhibitor BAY117082 and RR-L containing serum showed no better effect than BAY117082 along on MSCs in vitro. No differences were observed between RR-L containing serum and BAY117082 in (a) osteogenesis staining; (b) adipogenesis staining, and (c-e) NF-*κ*B signaling pathway activation of MSCs. (f–h) Statistical results of Alizarin red, Oil red staining, and IF analysis. (i) Representative western blots image of PPAR*γ*, RUNX2, OCN, p65, and *β*-actin. (j–m) Statistical results of western blot via Image Lab software. (n–q) mRNA expressions of PPAR*γ*, RUNX2, OCN, and p65. ^#^*p* < 0.05 versus ctrl, NS, *p* > 0.05 versus BAY117082 group.

To determine whether NF-*κ*B is the main signaling pathway of RR-L containing serum in regulating MSC differentiation, NF-*κ*B inhibitor BAY117082 was introduced in vitro. As shown in Figures 5(a)-5(b) and 5(f)-5(g), Alizarin red staining and Oil red staining results demonstrated that both cotreatment group (BAY117082 combined with RR-L containing serum) and BAY117082 group have a similar trend on osteogenesis promotion and adipogenesis inhibition on MSCs, and no significant differences were found between cotreatment group and BAY117082 group. Also, protein and mRNA expressions of differentiation biomarkers including PPAR*γ*, RUNX2, and OCN were detected, respectively. As shown in Figures 5(i)–5(q), gene and protein expressions of adipogenesis biomarker PPAR*γ* were significantly decreased in both the cotreatment group and the BAY117082 group. Meanwhile, gene and protein expressions of osteogenesis biomarker RUNX2 and OCN were significantly upregulated in both the cotreatment group and the BAY117082 group. No significant differences were found between the two groups. Figures 5(c)–5(e) and 5(h) illustrated that BAY117082 along significantly inhibited activation of NF-*κ*B, and no stronger effects were found in the cotreatment group. In conclusion, RR-L possibly altered differentiation behaviors of MSCs via downregulation of NF-*κ*B.

## 4. Discussion

In the current study, in the view of GO analysis, KEGG enrichment analysis, and hub gene analysis of “herb-compounds-disease” network of RR-L in treating OP, the results demonstrated that multiple inflammation signaling pathway and lipid metabolism-related signaling pathway are involved. For further understanding and validation of RR-L herb pair, OVX-induced OP rats were introduced as animal model in vivo. The results demonstrated that OVX-induced OP rats significantly manifested BMD loss and microarchitecture damage with abnormal activation of inflammation biomarkers and differentiation behaviors of MSCs. RR-L treatment reversed the trend which illustrated that RR-L herb pair is effective in treating OP and altering MSCs behaviors. To elucidate the molecular mechanism of RR-L in altering the MSCs differentiation, primary MSCs were collected and introduced in vitro. The results demonstrated that RR-L enhanced osteogenesis differentiation and suppressed adipogenesis differentiation of MSCs via downregulating the NF-*κ*B signaling pathway in a dose-dependent manner. Also, TNF-*α* and BAY117082 were introduced as activators and inhibitors of NF-*κ*B in vitro, respectively. The results demonstrated that once the TNF-*α*-induced NF-*κ*B pathway was activated, MSC differentiation behavior was altered and was similar to MSCs derived from OVX-induced OP rats. RR-L treatment reversed the trend via downregulation of NF-*κ*B. Also, with cotreatment of NF-*κ*B inhibitor, BAY117082 significantly obstructed the prominent effect of RR-L on MSCs, which illustrated that the NF-*κ*B signaling pathway is the crucial mechanism of RR-L in OP treatment.

OP, the most common metabolic bone disease, is characterized by BMD loss and microarchitecture damage, which leads to rising bone fracture risk. In vivo experiments in this study have demonstrated that RR-L treatment significantly improved BMD and restored damaged microarchitecture in OP rats, as shown in Figures 1(a)–1(d) and 1(j)-1(m). Also, MSCs obtained from these treated rats showed enhanced osteogenic differentiation potential compared to the untreated group, as shown in Figures 1(n)–1(o). Therefore, the RR-L could reverse OVX-induced OP phenotypes in rats via restoring the osteogenic potential of MSCs in vivo. Meanwhile, RR-L significantly downregulated NF-*κ*B nuclear translocation and downregulated serum inflammatory cytokines, indicating that this could possibly be the mechanism of RR-L on MSCs, as shown in Figures 1(g)–1(i) and 1(p)-1(t).

mscMSCs, a resident cell population existing in the bone marrow, are involved in the catabolic-anabolic coupling of bone metabolism [7, 23]. MSCs have multiple lineages of differentiation potential, including chondrogenesis, osteogenesis, adipogenesis, and stromal cells differentiation. Under physiological conditions, functional osteoclasts form on the bone surface, erode the bone matrix by dissolving the mineral components, and release the cytokines dormant in the bone matrix [24–26]. Released cytokines including TGF-*β* and other proosteogenesis cytokines [24, 26–28] recruited MSCs to the resorbing pit on the bone surface and induced the MSCs to differentiate into osteogenesis progenitor cells to repair the resorbed bone matrix [27]. The balance between osteogenesis and resorbing maintains the regular metabolism of bone. However, under pathological conditions of OP, bone-resorbing activity is abnormally upregulated and the MSCs differentiation potential is damaged, presented by massive osteoclasts formed and less MSCs differentiated into osteoblasts [7, 29]. Once the balance of bone formation and bone resorption fails, OP phenotypes occur, including bone mineral density loss, bone microarchitecture damage, and risk of fracture rise. At the level of cellular physiology, OP is characterized by damaged osteoblasts progenitor's formation and activities and abnormal activation and formation of osteoclasts. Currently, researchers had already developed efficient strategies, including first-line anti-OP drugs of bisphosphate and anti-RANKL antibody, on inhibiting abnormal osteoclasts formation and activity [3]. However, few compounds were successfully developed to promote the damaged differentiation behavior of MSCs or osteoblast progenitors. Hence, MSCs targeting strategy had attracted much researchers' attention for its pivotal role and therapeutical potential in OP. MSCs play a pivotal role in bone metabolism. Differentiation potential damage, mainly characterized by osteogenesis inhibition and adipogenesis upregulation, is the pathological characterization on the cellular level in OP progress. The current mainstream therapy on OP mainly focused on inhibiting abnormal bone resorption. The bone is under the dynamic balance between bone formation and resorption. The idea is that enhancing the MSC differentiation into osteoblast progenitors holds the key to enhancing bone formation. MSCs, as a resident cell population in the bone marrow, are with a limited number. Damaged differentiation potential impeded an adequate number of MSCs supplied into osteoblasts or osteoblast progenitors. Meanwhile, abnormally upregulated adipogenesis of MSCs resulted in an increasing level of inflammatory cytokines. In this study, RR-L has been proved to upregulate BMD and restore bone microarchitecture in vivo via enhancing the osteogenic differentiation potential of MSCs, which demonstrated that RR-L has the potential to be the alternative option in treating OP. In the current study, RR-L containing serum significantly increased MSC proliferation and enhanced osteogenic differentiation potential in a dose-dependent manner, along with upregulation of NF-*κ*B nuclear translocation of MSCs, as shown in Figure 3, which validated the efficacy of RR-L on MSCs.

Aberrant lineage differentiation of MSCs contributes to reduced bone mass, damaged microarchitecture, and increased bone marrow adipose tissue [7, 28, 29]. Although lineage alterations of MSCs have been identified to be the regulator of bone mass and structure, little is known about factors that are associated with osteoblastic differentiation and adipogenesis differentiation. It is well accepted that NF-*κ*B plays an important role in inflammation [30]. An NF-*κ*B signaling pathway is the main regulator of inflammation and involves multiple functions of inherent immunity and adaptive immunity. The activation of the NF-*κ*B signaling pathway is related to chronic inflammatory disorders, autoimmune diseases, and cancer [31, 32]. Targeting inhibition of NF-*κ*B transcription is a promising strategy in treatment. The NF-*κ*B signaling pathway is the key regulator which connects metabolism and inflammation. Previous studies had demonstrated that NF-*κ*B is the downstream effector of the great majority of stress signals induced by intracellular and extracellular metabolic disturbance [33]. OP is a bone metabolic disease. NF-*κ*B is also the main regulator involved in the OP. In the current study, RR-L manifested a promising strategy of regulating abnormally activation of NF-*κ*B to promote MSCs differentiated into osteoblasts. Here, the current studies identified NF-*κ*B signaling pathway as a crucial regulator of MSC fate decisions whose expressions increase with OP progressing. Activation of NF-*κ*B inhibited osteoblastic differentiation of MSCs and promoted adipogenesis differentiation, as shown in Figure 4. Conversely, inhibition of NF-*κ*B by BAY117082 repaired the damaged osteoblastic potentiation of MSCs and blocked MSCs differentiated into adipocytes, as shown in Figure 5. Mechanically, NF-*κ*B involves in bone formation and fat unbalance in OP progression. In general, RR-L exerts its anti-OP effects by restoring the differentiation potential of MSCs via inhibiting the NF-*κ*B signaling pathway.

## 5. Conclusion

To sum up, the results demonstrated the potentiation and mechanism of RR-L in treating OP. According to our findings, RR-L promoted osteogenesis differentiation and suppressed adipogenesis differentiation of MSCs via inhibiting of NF-*κ*B signaling pathway. The current study provided a new alteration of natural products in treating OP.

## Data Availability

All data generated or analyzed during this study are included within the article and supplementary file.

## Supplementary Materials

Supplementary Materials Supplementary Table 1: detailed chemical compound list of RR-L via network pharmacology analysis. Supplementary Table 2: 120 target genes of RR-L involved in treating OP. Supplementary Table 3: prime sequences. Supplementary Figure 1: the LC-MS analysis result of RR-L solution. All chromatographic peak signaling was recorded at 260 nm and the peak area was integrated under the instrument's protocol. Results demonstrated that different batches of RR-L solution were chemically stable. Peaks A and B, respectively, are identified as calycosin and liquiritin.
