# Supplementary material for: Shu-Di-Huang and Gan-Cao Herb Pair Restored the Differentiation Potentials of Mesenchymal Stem Progenitors in Treating Osteoporosis via Downregulation of NF-κB Signaling Pathway
Source: Evid Based Complement Alternat Med. 2021 Dec 14;2021:7795527. doi: 10.1155/2021/7795527 (PMC8692010; doi:10.1155/2021/7795527)
Supplement: Supplementary Materials — Supplementary Table 1: detailed chemical compound list of RR-L via network pharmacology analysis. Supplementary Table 2: 120 target genes of RR-L involved in treating OP. Supplementary Table 3: prime sequences. Supplementary Figure 1: the LC-MS analysis result of RR-L solution. All chromatographic peak signaling was recorded at 260 nm and the peak area was integrated under the instrument's protocol. Results demonstrated that different batches of RR-L solution were chemically stable. Peaks A and B, respectively, are identified as calycosin and liquiritin. [file 7795527.f1.docx]

**Supplementary Table 1** Detailed chemical compounds list of RR-L via network pharmacology analysis

| chemical compounds of RR-L |
| --- |
| Arachic acid |
| EIC |
| PENTADECYLIC ACID |
| leonuride |
| Daturic acid |
| acteoside |
| methyl (2E,4E)-hexadeca-2,4-dienoate |
| zoomaric acid |
| Sumiki's acid |
| Dihydro-beta-ionone |
| Pca |
| catalpol |
| catapol_qt |
| MTL |
| lauric acid |
| 8-epi-Loganic acid |
| Forsythiaside |
| succinic acid |
| Sitogluside |
| sitosterol |
| FER |
| 2-(4-hydroxyphenyl)ethyl hexacosanoate |
| aeginetic acid |
| Ajugol |
| Ajugoside |
| Ajugoside_qt |
| Cerebrosid |
| Cistanoside A |
| Cistanoside F |
| methyl 9,10-methylene-hexadecanoate |
| (2S,3R,4R,5S,6R)-2-[[(1S,4aS,5R,7aR)-4a,5-dihydroxy-7-methylol-5,7a-dihydro-1H-cyclopenta[c]pyran-1-yl]oxy]-6-methylol-tetrahydropyran-3,4,5-triol |
| melittoside_qt |
| Dihydrocatalpol |
| geniposide |
| geniposide_qt |
| glutinoside |
| Jiofuran |
| jioglutin A |
| jioglutin B |
| jioglutin C |
| jioglutin D |
| jioglutin E |
| jioglutolide |
| Jioglutoside A |
| methyl (1S,4aS,7aS)-7-methylene-1-[(2S,3R,4S,5S,6R)-3,4,5-trihydroxy-6-[[(2R,3R,4R,5S,6R)-3,4,5-trihydroxy-6-methyl-tetrahydropyran-2-yl]oxymethyl]tetrahydropyran-2-yl]oxy-4a,5,6,7a-tetrahydro-1H-cyclopenta[d]pyran-4-carboxylate |
| Jioglutoside B_qt |
| Jionoside A |
| Jionoside B |
| Melittoside |
| METHYL PALMITOLEATE |
| 6-O-p-coumaroylajugol |
| methyl-2,6,10-trimethyltridecanoate |
| Purpureaside C |
| Rehmaglutin B |
| (3aS,4R,6aS)-4-hydroxy-6,6a-dimethylol-3a,4-dihydro-3H-cyclopenta[d]furan-2-one |
| (2S,3R,4S,5S,6R)-2-[(1R,2R)-2-hydroxy-2-[(E,3S)-3-hydroxybut-1-enyl]-1,3,3-trimethylcyclohexoxy]-6-(hydroxymethyl)tetrahydropyran-3,4,5-triol |
| (2S,3R,4S,5S,6R)-2-[(1R,2R)-2-hydroxy-2-[(E,3R)-3-hydroxybut-1-enyl]-1,3,3-trimethylcyclohexoxy]-6-(hydroxymethyl)tetrahydropyran-3,4,5-triol |
| Rehmaionoside C |
| rehmannioside B |
| rehmannioside C |
| 6-O-p-hydroxybenzoylajugol |
| (3R)-2,6,6-trimethyl-3-[(2R,3R,4S,5S,6R)-3,4,5-trihydroxy-6-(hydroxymethyl)oxan-2-yl]oxycyclohexene-1-carboxylic acid |
| Rehmannioside A |
| Rehmaglutin A |
| Rehmaglutin D |
| 6-O-vanilloylajugol |
| echinacoside |
| aucubin |
| Acetylcatalpol |
| Docosanoate |
| Caffeate |
| Stigmasterol |
| Stachyose |
| HMF |
| raffinose |
| sucrose |
| Inermine |
| DFV |
| Mairin |
| Glycyrol |
| Jaranol |
| Medicarpin |
| isorhamnetin |
| sitosterol |
| Lupiwighteone |
| 7-Methoxy-2-methyl isoflavone |
| formononetin |
| Calycosin |
| kaempferol |
| naringenin |
| (2S)-2-[4-hydroxy-3-(3-methylbut-2-enyl)phenyl]-8,8-dimethyl-2,3-dihydropyrano[2,3-f]chromen-4-one |
| euchrenone |
| glyasperin B |
| glyasperin F |
| Glyasperin C |
| Isotrifoliol |
| (E)-1-(2,4-dihydroxyphenyl)-3-(2,2-dimethylchromen-6-yl)prop-2-en-1-one |
| kanzonols W |
| (2S)-6-(2,4-dihydroxyphenyl)-2-(2-hydroxypropan-2-yl)-4-methoxy-2,3-dihydrofuro[3,2-g]chromen-7-one |
| Semilicoisoflavone B |
| Glepidotin A |
| Glepidotin B |
| Phaseolinisoflavan |
| Glypallichalcone |
| 8-(6-hydroxy-2-benzofuranyl)-2,2-dimethyl-5-chromenol |
| Licochalcone B |
| licochalcone G |
| 3-(2,4-dihydroxyphenyl)-8-(1,1-dimethylprop-2-enyl)-7-hydroxy-5-methoxy-coumarin |
| Licoricone |
| Gancaonin A |
| Gancaonin B |
| licorice glycoside E |
| 3-(3,4-dihydroxyphenyl)-5,7-dihydroxy-8-(3-methylbut-2-enyl)chromone |
| 5,7-dihydroxy-3-(4-methoxyphenyl)-8-(3-methylbut-2-enyl)chromone |
| 2-(3,4-dihydroxyphenyl)-5,7-dihydroxy-6-(3-methylbut-2-enyl)chromone |
| Glycyrin |
| Licocoumarone |
| Licoisoflavone |
| Licoisoflavone B |
| licoisoflavanone |
| shinpterocarpin |
| (E)-3-[3,4-dihydroxy-5-(3-methylbut-2-enyl)phenyl]-1-(2,4-dihydroxyphenyl)prop-2-en-1-one |
| liquiritin |
| licopyranocoumarin |
| 3,22-Dihydroxy-11-oxo-delta(12)-oleanene-27-alpha-methoxycarbonyl-29-oic acid |
| Glyzaglabrin |
| Glabridin |
| Glabranin |
| Glabrene |
| Glabrone |
| 1,3-dihydroxy-9-methoxy-6-benzofurano[3,2-c]chromenone |
| 1,3-dihydroxy-8,9-dimethoxy-6-benzofurano[3,2-c]chromenone |
| Eurycarpin A |
| glycyroside |
| (-)-Medicocarpin |
| Sigmoidin-B |
| (2R)-7-hydroxy-2-(4-hydroxyphenyl)chroman-4-one |
| (2S)-7-hydroxy-2-(4-hydroxyphenyl)-8-(3-methylbut-2-enyl)chroman-4-one |
| Isoglycyrol |
| Isolicoflavonol |
| HMO |
| 1-Methoxyphaseollidin |
| Quercetin der. |
| 3'-Hydroxy-4'-O-Methylglabridin |
| licochalcone a |
| 3'-Methoxyglabridin |
| 2-[(3R)-8,8-dimethyl-3,4-dihydro-2H-pyrano[6,5-f]chromen-3-yl]-5-methoxyphenol |
| Inflacoumarin A |
| icos-5-enoic acid |
| Kanzonol F |
| 6-prenylated eriodictyol |
| 7,2',4'-trihydroxy－5-methoxy-3－arylcoumarin |
| 7-Acetoxy-2-methylisoflavone |
| 8-prenylated eriodictyol |
| gadelaidic acid |
| Vestitol |
| Gancaonin G |
| Gancaonin H |
| Licoagrocarpin |
| Glyasperins M |
| Glycyrrhiza flavonol A |
| Licoagroisoflavone |
| 18α-hydroxyglycyrrhetic acid |
| Odoratin |
| Phaseol |
| Xambioona |
| dehydroglyasperins C |
| quercetin |

**Supplementary Table 2** 120 target genes of RR-L involving treating OP

| Target genes |
| --- |
| PTGS1 |
| PTGS2 |
| F7 |
| RXRA |
| ADRB2 |
| ESR1 |
| SLC6A4 |
| PGR |
| NOS2 |
| PPARG |
| KDR |
| MAPK14 |
| GSK3B |
| CCNA2 |
| AR |
| ESR2 |
| ACHE |
| RELA |
| OLR1 |
| NR3C2 |
| ADRB1 |
| JUN |
| IL4 |
| HSD3B2 |
| HSD3B1 |
| AKT1 |
| BCL2 |
| BAX |
| CASP3 |
| MAPK8 |
| MMP1 |
| STAT1 |
| HMOX1 |
| CYP3A4 |
| CYP1A2 |
| CYP1A1 |
| ICAM1 |
| SELE |
| VCAM1 |
| NR1I2 |
| CYP1B1 |
| ALOX5 |
| GSTP1 |
| AHR |
| SLC2A4 |
| NR1I3 |
| INSR |
| GSTM1 |
| SLPI |
| MAPK3 |
| MAPK1 |
| FASN |
| LDLR |
| SOD1 |
| CAT |
| APOB |
| HMGCR |
| UGT1A1 |
| PPARA |
| SREBF1 |
| GSR |
| ADIPOQ |
| AKR1C1 |
| STAT3 |
| CCND1 |
| MMP3 |
| EGFR |
| VEGFA |
| BCL2L1 |
| FOS |
| CDKN1A |
| CASP9 |
| PLAU |
| MMP2 |
| MMP9 |
| IL10 |
| EGF |
| IL6 |
| TP63 |
| HIF1A |
| ERBB2 |
| CAV1 |
| MYC |
| F3 |
| IL1B |
| CCL2 |
| CXCL8 |
| BIRC5 |
| NOS3 |
| HSPB1 |
| SULT1E1 |
| IL2 |
| PLAT |
| THBD |
| SERPINE1 |
| COL1A1 |
| IFNG |
| IL1A |
| MPO |
| ABCG2 |
| NFE2L2 |
| NQO1 |
| PARP1 |
| COL3A1 |
| PPARD |
| CRP |
| SPP1 |
| RUNX2 |
| CTSD |
| IGFBP3 |
| IGF2 |
| CD40LG |
| IRF1 |
| PON1 |
| NR3C1 |
| CD80 |
| CD40 |
| GLA |
| LCT |
| CHI3L1 |

Supplementary Table 3 Prime Sequences

| Gene | Forward | Reverse |
| --- | --- | --- |
| PPARγ | 5'-TTTTCAAGGGTGCCAGTTTC-3' | 5'-AATCCTTGGCCCTCTGAGAT-3' |
| RUNX2 | 5'-CCGCACGACAACCGCACCAT-3' | 5'- CGCTCCGGCCCACAAATCTC-3' |
| OCN/BGLAP | 5'- CAGCCACCGAGACACCAT-3' | 5'- CCAGCAGAGCGACACCCTA-3' |
| p65/RELA | 5'-GAGACATCCTTCCGCAAACT-3' | 5'-GGAGATTACTGCCCTGGCTCCTA-3' |


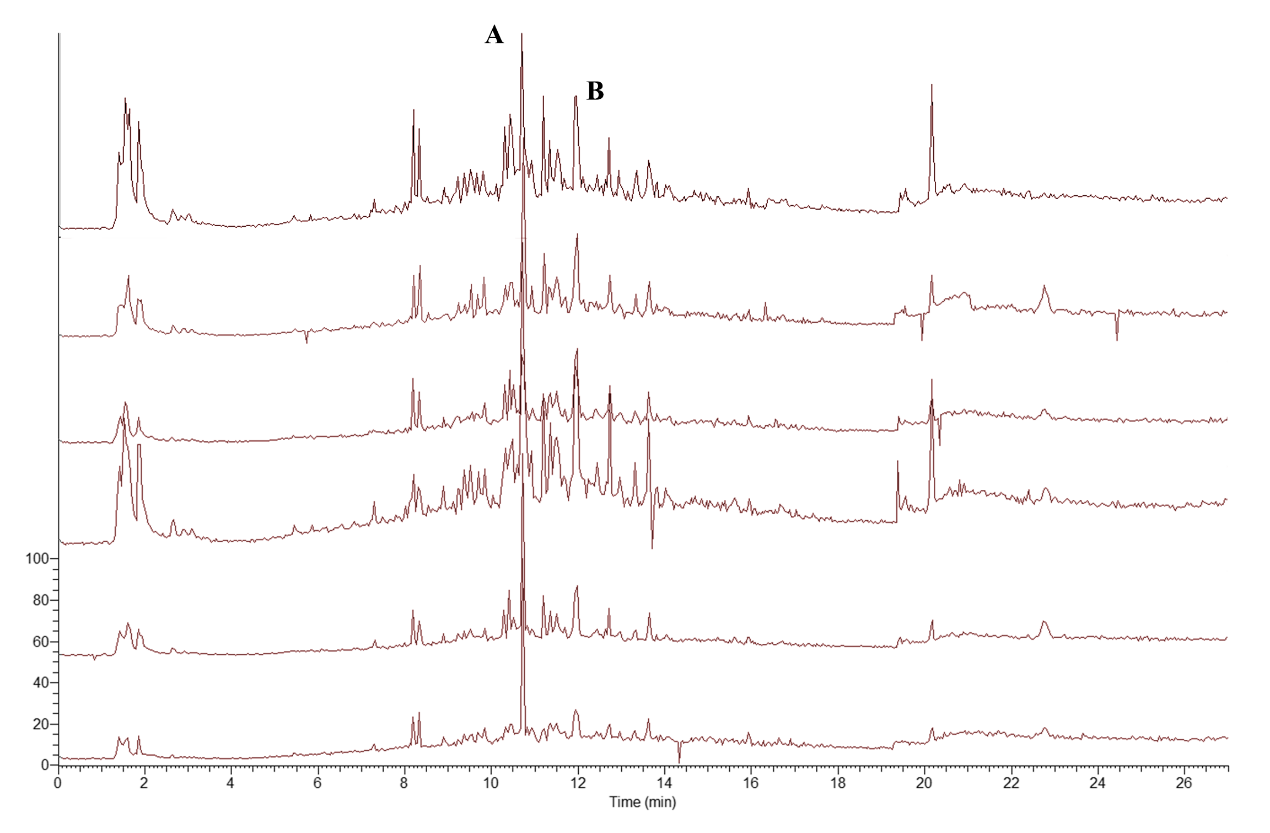


**Supplementary Figure 1**. The LC-MS analysis result of RR-L solution. All chromatographic peak signalling was recorded at 260 nm and the peak area was integrated under the instrument’s protocol. Results demonstrated that different batches of RR-L solution were chemically stable. Peak A and B respectively identified as calycosin and Liquiritin.
